# High glucose-induced oxidative stress impairs proliferation and migration of human gingival fibroblasts

**DOI:** 10.1371/journal.pone.0201855

**Published:** 2018-08-09

**Authors:** Prima Buranasin, Koji Mizutani, Kengo Iwasaki, Chantida Pawaputanon Na Mahasarakham, Daisuke Kido, Kohei Takeda, Yuichi Izumi

**Affiliations:** 1 Department of Periodontology, Graduate School of Medical and Dental Sciences, Tokyo Medical and Dental University (TMDU), Tokyo, Japan; 2 Department of Nanomedicine, Graduate School of Medical and Dental Sciences, Tokyo Medical and Dental University (TMDU), Tokyo, Japan; 3 Department of Restorative Dentistry, Faculty of Dentistry, Khon Kaen University, Khon Kaen, Thailand; University of South Alabama Mitchell Cancer Institute, UNITED STATES

## Abstract

Delayed gingival wound healing is widely observed in periodontal patients with diabetes. However, the molecular mechanisms of the impaired function of gingival fibroblasts in diabetes remain unclear. The purpose of this study was to investigate changes in the properties of human gingival fibroblasts (HGFs) under high-glucose conditions. Primary HGFs were isolated from healthy gingiva and cultured with 5.5, 25, 50, and 75 mM glucose for 72 h. *In vitro* wound healing, 5-ethynyl-2′-deoxyuridine (EdU), and water-soluble tetrazolium salt (WST-8) assays were performed to examine cell migration and proliferation. Lactase dehydrogenase (LDH) levels were measured to determine cytotoxicity. The mRNA expression levels of oxidative stress markers were quantified by real-time PCR. Intracellular reactive oxygen species (ROS) were also measured in live cells. The antioxidant *N*-acetyl-l-cysteine (NAC, 1 mM) was added to evaluate the involvement of ROS in the glucose effect on HGFs. As a result, the *in vitro* wound healing assay showed that high glucose levels significantly reduced fibroblast migration and proliferation at 6, 12, 24, 36, and 48 h. The numbers of cells positive for EdU staining were decreased, as was cell viability, at 50 and 75 mM glucose. A significant increase in LDH was proportional to the glucose concentration. The mRNA levels of heme oxygenase-1 and superoxide dismutase-1 and ROS levels were significantly increased in HGFs after 72 h of exposure to 50 mM glucose concentration. The addition of NAC diminished the inhibitory effect of high glucose in the *in vitro* wound healing assay. The results of the present study show that high glucose impairs the proliferation and migration of HGFs. Fibroblast dysfunction may therefore be caused by high glucose-induced oxidative stress and may explain the delayed gingival wound healing in diabetic patients.

## Introduction

Diabetes is a metabolic disease characterized by increased blood glucose levels. The impaired metabolism of glucose, lipids, and proteins produces alterations in macro- and microvascular circulation, giving rise to the risk of several complications in patients with diabetes, including retinopathy, neuropathy, nephropathy, cardiovascular complications [1], and delayed wound healing [2]. Periodontal disease is a chronic inflammatory disease of the tissues that support and attach the teeth to the jaws. An abundance of evidence suggests a relationship between diabetes and periodontal disease [3]. Many studies in various populations have demonstrated that diabetic patients tend to have a higher prevalence of and more severe periodontitis than nondiabetics [4].

Hyperglycemia, a key abnormality in diabetes, plays an important role in the development of inflammation in diabetic complications. It has been demonstrated that high blood sugar promotes inflammation and inhibits wound healing by altering angiogenesis [5]. In *in vitro* studies, hyperglycemia has been shown to reduce migration [6, 7], proliferation [8], and collagen synthesis [9] and increase apoptosis [10, 11] in various cell types.

Wound healing requires the complex coordination of several cell types, including keratinocytes, fibroblasts, endothelial cells, macrophages, and platelets. Successful wound healing is accomplished by a series of coordinated processes that include cell migration and proliferation, collagen deposition and remodeling, and wound contraction and angiogenesis. Fibroblasts are the most abundant cell type in connective tissue and are involved in producing and remodeling the extracellular matrix; hence, they have an important role in gingival breakdown [9]. The responses of gingival fibroblasts to elevated concentrations of glucose presumably play an essential role in the wound healing of periodontal tissue in diabetic patients [12]. Furthermore, unfavorable soft tissue regeneration and healing responses in patients with poorly controlled diabetes are known complications after periodontal therapy and oral surgery [13, 14].

Over time, oxidative stress can be an important pathogenic factor in diabetic complications. Patients with diabetes have elevated levels of advanced glycation end products in their gingival tissues that may be associated with a state of enhanced oxidative stress, a potential mechanism for accelerated tissue damage [15]. Moreover, oxidative stress may be a key mechanism underlying the increase in cellular abnormalities in susceptible individuals [16]. Various biologically plausible mechanisms have been proposed for a common inflammatory etiopathogenesis between diabetes and periodontal disease. However, the underlying molecular mechanisms are still controversial and may even be distinct in different cell types.

With the aim of elucidating the relationship between periodontal disease and diabetes, the purpose of this *in vitro* study was to investigate the effect of high glucose on changes in human gingival fibroblast (HGFs) migration and proliferation.

## Materials and methods

### Primary cell culture

Gingival connective tissue samples were obtained from systemically healthy patients during gingivectomy, crown lengthening, or a connective tissue graft procedure in the Department of Periodontology at the Dental Hospital of Tokyo Medical and Dental University (TMDU). The study protocol was approved by the ethical committee of the Faculty of Dentistry, TMDU (D2014-092-01), and written inform consent for participation in the study was obtained from each subject. In brief, gingival specimens were taken from the non-inflamed periodontal tissues of one male (62 years old) and two females (27 and 68 years old) who represented systemically healthy, non-smoking donors. All the subjects had received periodontal therapy consisting of a full-mouth scaling and root planning, without the use of systemic antibiotics within 3 months before the surgery. Gingival tissue was washed in phosphate-buffered saline (PBS) containing 3% antibiotics and immersed in Dulbecco’s modified Eagle’s medium (D-MEM; Wako, Osaka, Japan) containing 20% dispase I for 24 h at 4°C to separate the connective tissue from the epithelial layers [17]. The connective tissue was minced into small pieces (1 mm^2^) and placed in a 10-cm tissue culture dish in D-MEM supplemented with 10% fetal bovine serum (FBS, Biosera, Miami, FL, USA) and 1% antibiotic-antimycotic mixture (Gibco, Grand Island, NY, USA). Tissue was then incubated at 37°C under a humidified atmosphere of 95% air and 5% CO_2_. HGFs from passages 3–6 were used for experiments. Thereafter, HGFs were grown in normal medium (5.5 mM glucose) and three high-glucose (d-glucose) media (high glucose; hyperglycemic media) containing 25, 50, or 75 mM d-glucose for 72 h prior to the experiments. For osmotic control, mannitol was added as a control for hyperosmolarity in the high-glucose groups [7].

### *In vitro* wound healing assay

To determine the optimal glucose concentration in cultured HGFs, an *in vitro* scratch assay was performed. Cells were plated on uncoated 6-well culture dishes at a density of 6 × 10^4^ cells/well and incubated in 5.5 mM glucose until 80% confluence. Cells were then exposed to each glucose concentration. A scratch was manually made across the cell monolayer using a p200 pipette tip (Thermo Fisher Scientific, Waltham, MA, USA), removing cells from an area with a width of 1.5 ± 0.5 mm. Cells were then washed with PBS to remove cellular debris, and fresh medium was replaced immediately. After scratching, cells were observed under a phase-contrast microscope until the wound healed in all the groups, and digital photographs were taken by matching reference points. The percent cell migration and the area under the percent migration curve (AUC) were calculated using ImageJ software (National Institutes of Health, Bethesda, MD, USA).

### Cell proliferation

Cell proliferation rates were determined by the uptake of 5-ethynyl-2′-deoxyuridine (EdU) into DNA using a Click-iT EdU microplate assay kit (Invitrogen, Carlsbad, CA, USA) according to the manufacturer’s instructions. The fluorescence at 490 nm (excitation)/585 nm (emission) was measured with a 2030 ARVO MX microplate reader and expressed as the cell proliferation rate. HGFs were seeded in 96-well plates at a density of 4 × 10^3^ cells/well and incubated in 5.5 mM glucose until 80% confluence. Cells were cultured under each glucose concentration for 72 h. Then, 10 μl of EdU solution was added to each well, and the plate was incubated for 18 h. After nuclear staining with 1 mg/ml Hoechst 33342 (Dojindo, Kumamoto, Japan) for 30 min at room temperature, the cells were observed under a Keyence fluorescence microscope BZ-8000 (Keyence, Osaka, Japan). The percentage of EdU-positive cells was determined by counting EdU-positive nuclei and total nuclei in 10 randomly captured fields per well.

### Cell proliferation and lactate dehydrogenase (LDH) assays

Cell proliferation was also determined with a water-soluble tetrazolium salt (WST-8) colorimetric assay using Cell Counting Kit-8 (Dojindo). After culturing the cells under each glucose concentration for 72 h, the culture medium was completely removed from the samples and freshly prepared culture medium was added containing 1 v% WST-8 assay kit, prior to incubation for 30 min. Subsequently, 100 μl of supernatant from each sample was transferred into a well of a 96 well-plate, and absorbance was measured at 450 nm with a microplate reader.

LDH levels were measured using a Cytotoxicity Detection Kit (Roche, Mannheim, Germany) according to the manufacturer’s instructions. The results were assessed by measuring optical absorbance using a microplate reader at 490 nm.

### Real-time polymerase chain reaction (PCR)

Total RNA was extracted with an RNeasy Mini Kit (Qiagen Inc., Valencia, CA, USA). RNA concentrations were quantified by NanoDrop Lite (Thermo Fisher Scientific). cDNA was synthesized using a PrimeScript RT Reagent Kit (Takara, Shiga, Japan). Real-time PCR was performed using SYBR Premix Ex Taq^™^ II (Takara) with a Thermal Cycler Dice Real Time System II (Takara). The mRNA levels of the oxidative markers nuclear factor erythroid 2 (NFE2)-related factor 2 (*NRF2)*, heme oxygenase 1 *(HO1)*, superoxide dismutase 1 *(SOD1)*, and catalase (*CAT*) were quantified. β-actin was used as a control to determine relative gene expression levels. Primer sequences are listed in S1 Table.

### Reactive oxygen species (ROS) production

To assess the generation of high glucose-mediated oxidative stress in live cells cultured under high glucose concentrations, intracellular ROS levels were measured using the ROS-ID^®^ Hypoxia/Oxidative Stress Detection kit (Enzo Life Sciences, Farmingdale, NY, USA). HGFs were cultured in a 96 well-plate with 4 × 10^3^ cells/well and were grown in complete medium until reaching 80% confluence before treatment. Cells were treated with each glucose concentration for 72 h and then treated with the kit reagent mix according to the manufacturer’s instructions. Cell nuclei were stained with 1 mg/ml Hoechst 33342 and observed by fluorescence microscope. Data were obtained from 10 randomly captured fields per well using ImageJ software.

### Antioxidant treatment

The cysteine prodrug *N*-acetyl-l-cysteine (NAC) is widely used as a pharmacological antioxidant and cytoprotectant. It has been reported to lower endogenous oxidant levels and to protect cells against a wide range of pro-oxidative insults [18]. NAC itself can serve as an antioxidant by reacting directly with free radicals [19]. In order to investigate whether NAC could reduce high glucose-induced cytotoxicity by decreasing ROS levels, cells were treated with each glucose concentration in the presence and absence of NAC. To determine the optimal concentration of NAC (Sigma-Aldrich, St. Louis, MO, USA), various concentrations of NAC (100 μM, 500 μM, and 1 mM) were applied to HGFs in control and high glucose (50 mM) groups in the scratch assay. Following this, to confirm the ability of NAC to reduce the effect of high glucose-induced toxicity on cell proliferation, HGFs at all glucose concentrations were treated with or without 1 mM NAC and the EdU assay was performed as described above. HGFs were cultured with 5.5 mM glucose medium in a 96-well plate and pretreated with 1 mM NAC at 80% confluence. After 24 h, cells were then exposed to each glucose concentration for 72 h, followed by the EdU assay. Similarly, the WST-8 test was also performed as described above.

From studies conducted on various cell types, it appears that NAC possesses both growth-promoting and anti-apoptotic activities. To determine whether NAC has the potential to inhibit cell damage in HGFs, LDH levels were measured after cells were treated with 1 mM NAC, with the same protocol as previously described above.

### Statistical analysis

Data are presented as means ± standard deviations (SDs) obtained from three independent treatments (biological replicates). Differences between groups were analyzed by one-way analysis of variance (ANOVA) followed by the Tukey-Kramer post hoc test and accepted as statistically significant at *p* < 0.05.

## Results

### High glucose impaired migration and proliferation in HGFs

#### Cell migration

**Fig 1:**
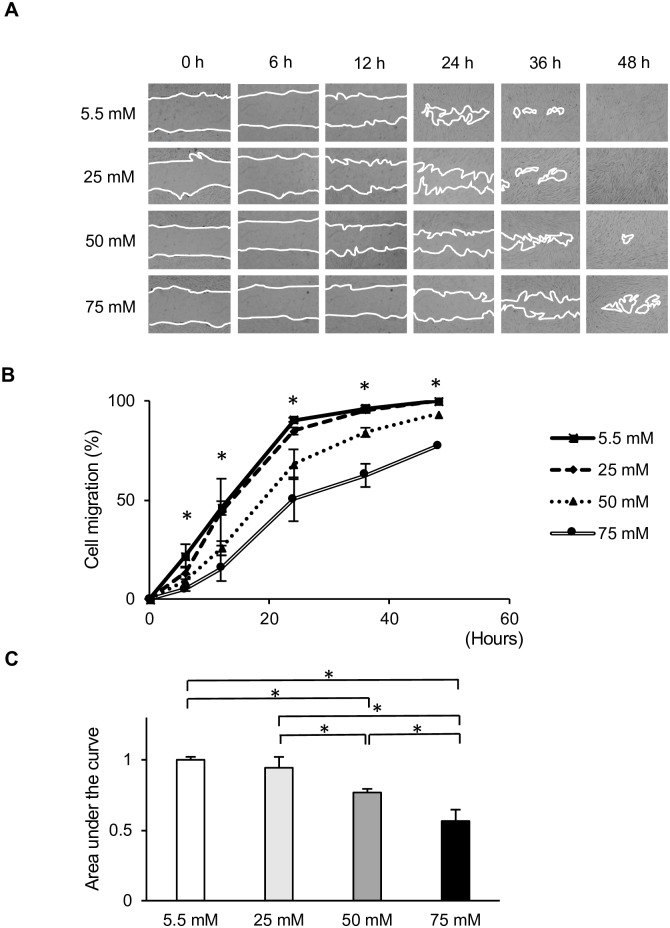
*In vitro* wound healing assay at various glucose concentrations. The effects of low and high glucose levels on human gingival fibroblast (HGFs) migration were assessed using wound healing assays. (A) Representative photomicrographs show cell migration at 0, 6, 12, 24, 36, and 48 h. Magnification ×40. (B) Relative cell migration areas of HGFs cultured at various glucose concentrations. (C) Area under the curve (AUC) of the relative cell migration area of HGFs cultured at various glucose concentrations. Data are presented as means ± SD of three independent experiments; mean differences between groups were analyzed using the Tukey–Kramer test. *p < 0.05.

To analyze the molecular mechanisms underlying the delayed wound healing of gingival tissues in diabetic patients, we first examined the effect of increased glucose levels on HGFs migration *in vitro* using a wound healing assay. As shown in Fig 1A and 1B, HGFs migration was significantly inhibited in cells cultured at higher glucose levels (50 and 75 mM), resulting in prolonged wound closure. This inhibition was observed within 6 h and lasted up to at least 48 h. In contrast, the migration of cells incubated at 25 mM glucose was only slightly slower than that of the control (5.5 mM) group. The AUC of the relative cell migration area from each group was calculated. The AUC changed depending on the concentration of glucose in the culture medium (Fig 1C). HGFs cultured at 50 and 75 mM glucose showed significantly slower migration than those cultured at normal glucose levels. Cell migration and wound closure was the slowest in cells grown at 75 mM glucose.

#### Cell proliferation

**Fig 2:**
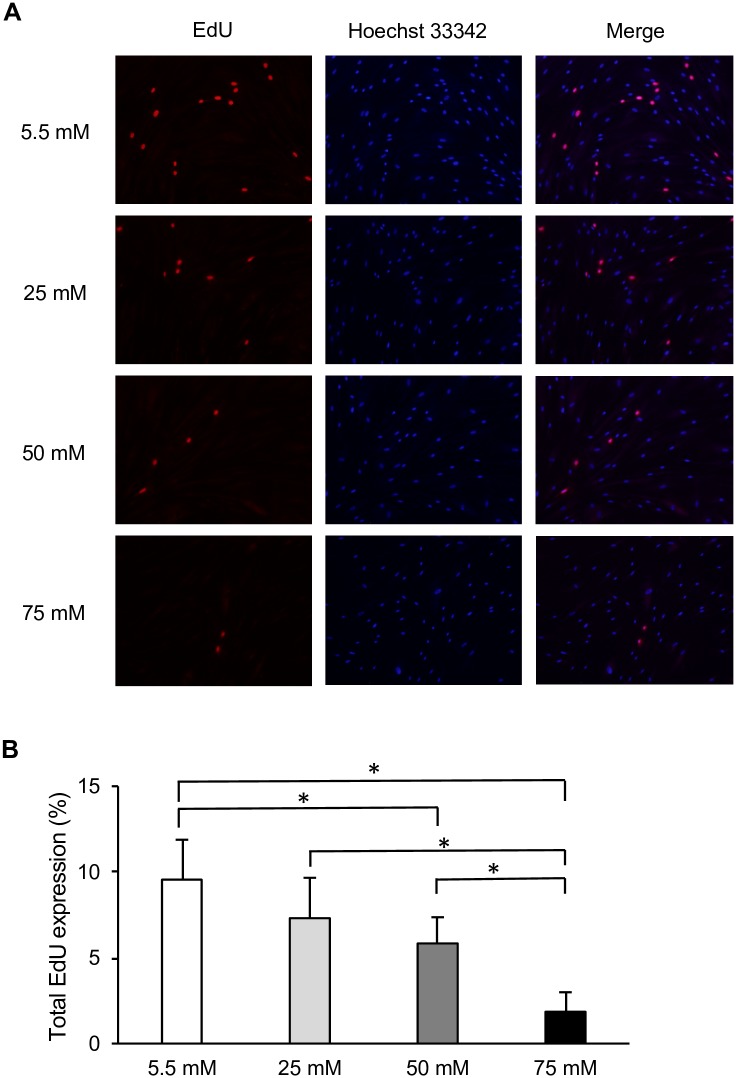
High glucose inhibited human gingival fibroblast cell proliferation. Proliferation was measured in cells at various glucose concentrations by 5-ethynyl-2′-deoxyuridine (EdU) incorporation assay. (A) Representative immunofluorescence images of EdU incorporated into mitochondrial DNA, Hoechst 33342 staining, and merged images. (B) Quantitative analysis of EdU-positive cells. Data are presented as means ± SD of three independent experiments; mean differences between groups were analyzed using the Tukey–Kramer test. *p < 0.05.

**Fig 3:**
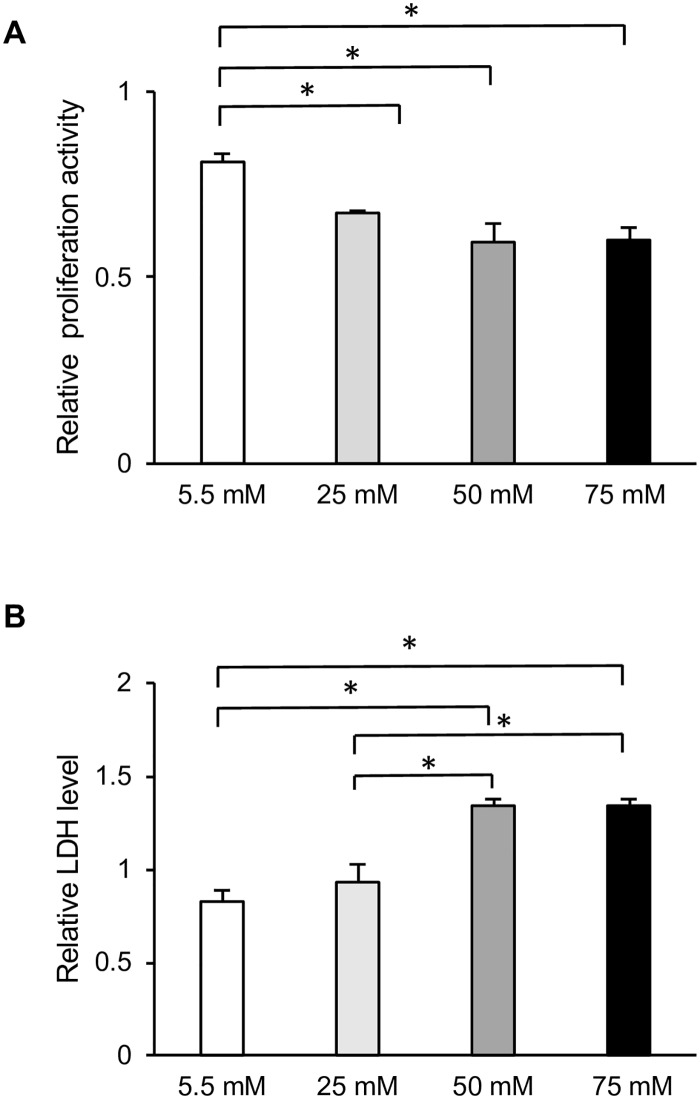
High glucose inhibited cell proliferation and induced cell death in human gingival fibroblasts (HGFs). (A) Water-soluble tetrazolium salt (WST-8) proliferation assay was performed after 72-h culture of HGFs with the indicated concentrations of glucose. (B) Lactate dehydrogenase (LDH) cytotoxicity assay was performed after 72-h culture of HGFs with the indicated concentrations of glucose. Data are expressed as mean values ± SD; mean differences between groups were analyzed using the Tukey–Kramer test *p < 0.05 compared to values in control group.

To investigate the involvement of cell proliferation in delayed wound closure, we next examined the effect of different concentrations of glucose on cell growth. EdU incorporation assays were performed to investigate the growth of HGFs at different glucose concentrations. After 72 h of incubation, the percentage of EdU-positive cells was decreased by higher glucose levels in a dose-dependent manner, as demonstrated in Fig 2A and 2B. Among all glucose concentrations, cell proliferation at 50 and 75 mM glucose was significantly reduced compared with that in the control group (5.5 mM). As with cell migration, cell proliferation was lowest among HGFs grown at 75 mM glucose (Fig 2A and 2B). WST-8 assays were also performed to confirm the effect of high glucose on HGFs proliferation. No significant differences were observed between the low and high glucose groups after 6, 24, or 48 h of incubation (S1 Fig). However, after 72 h, the absorbance values tended to decrease with higher glucose concentrations in a dose-dependent manner, as shown in Fig 3A. This decrease was also observed in the osmotic control cells, in which mannitol was added to maintain the same osmolarity as that observed under conditions of high glucose.

#### Cell cytotoxicity

The release of LDH into the medium is a hallmark of cytotoxicity. After 72 h of exposure to high glucose, LDH levels in the supernatant were directly proportional to the glucose concentration. The incubation of HGFs with 50 and 75 mM glucose resulted in a significant increase in cellular damage compared with that in the control group. In contrast, incubation with 25 mM glucose did not induce any significant change in LDH levels (Fig 3B). LDH levels indicative of cellular damage were also induced by the addition of mannitol at a concentration needed to maintain the same osmolarity as in the high-glucose group.

### High glucose increased ROS production in HGFs

#### mRNA expression of oxidative stress markers

**Fig 4:**
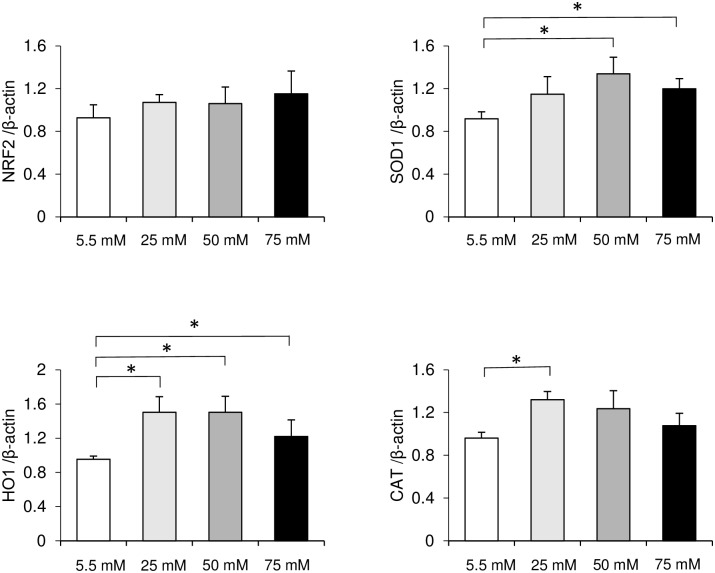
Oxidative stress marker gene expression. The mRNA expression levels of (A) *NRF2*, (B) *HO1*, (C) *SOD1*, and (D) *CAT* were quantified by real-time polymerase chain reaction (PCR) in human gingival fibroblasts grown at different glucose concentrations for 72 h. Data are expressed as mean values ± SD; mean differences between groups were analyzed using the Tukey–Kramer test. *p < 0.05.

ROS production is a major regulator in diabetic disease. To investigate whether high glucose affects the mRNA expression of oxidative markers, gene expression levels were determined by real-time PCR. The expression of the anti-oxidative markers *NRF2*, *HO1*, *SOD1*, and *CAT* were up-regulated in the high-glucose groups. Elevations in *NRF2* gene expression were proportional to increases in glucose in a concentration-dependent manner. However, no significant differences were found within samples at each glucose concentration (Fig 4A). mRNA levels of *HO1* and *SOD1* were highest in the 50 mM glucose group and significantly higher than those in the control group (Fig 4B and 4C). In contrast, mRNA levels of *CAT* were the highest in the 25 mM glucose group and significantly higher than those in the control group (Fig 4D). These data suggest that high glucose produces oxidative stress in HGFs.

#### Intracellular ROS

**Fig 5:**
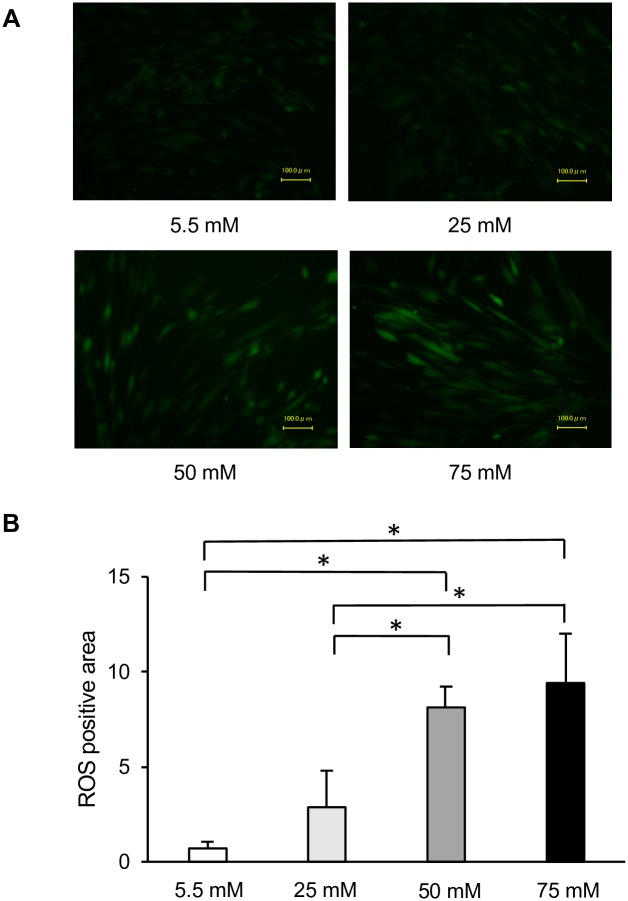
Measurement of intracellular reactive oxygen species (ROS) levels. Production of ROS in cultured human gingival fibroblasts in the presence of constant high-glucose levels for 72 h. (A) A fluorogenic probe for oxidative stress levels was detected using an appropriate filter for fluorescein. Images were acquired using 10x magnification and are representative of three different experiments. (B) Quantification of data shown in A. Data are expressed as mean values ± SD; mean differences between groups were analyzed using the Tukey–Kramer test. *p < 0.05.

Next, we measured ROS accumulation to determine the effect of high glucose on oxidative stress in HGFs. As shown in Fig 5A, the upregulation of ROS was detectable at 72 h after induction of high-glucose conditions. The intracellular ROS levels also increased proportionally to the glucose concentration. Significant increases in ROS accumulation in HGFs were observed after exposure to both 50 and 75 mM glucose. However, quantitative analyses revealed no significant differences between ROS production between 50 and 75 mM glucose groups (Fig 5B). Taken together, these data confirm the involvement of ROS in high glucose-induced impairments in HGFs cell migration and proliferation and in high glucose-induced toxicity.

### Role of antioxidant defense system and protection mechanism

**Fig 6:**
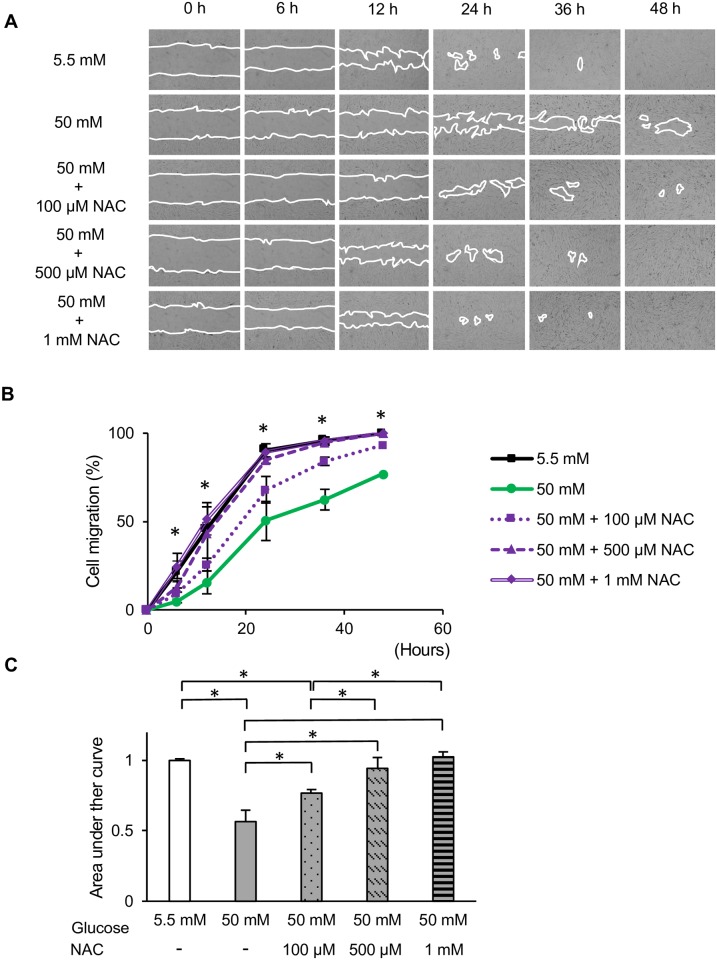
*In vitro* wound healing assay in the presence of *N*-acetyl-l-cysteine (NAC). Effects of the antioxidant NAC on high glucose-induced toxicity in human gingival fibroblasts in the absence and presence of NAC (100 μM, 500 μM, and 1 mM) were measured using *in vitro* wound healing assays. (A, B) Representative photomicrographs and relative cell migration areas. Magnification ×40. (C) The area under the curve (AUC) of the relative cell migration area. Data are presented as means ± SD of three independent experiments; mean differences between groups were analyzed using the Tukey–Kramer test. *p < 0.05.

**Fig 7:**
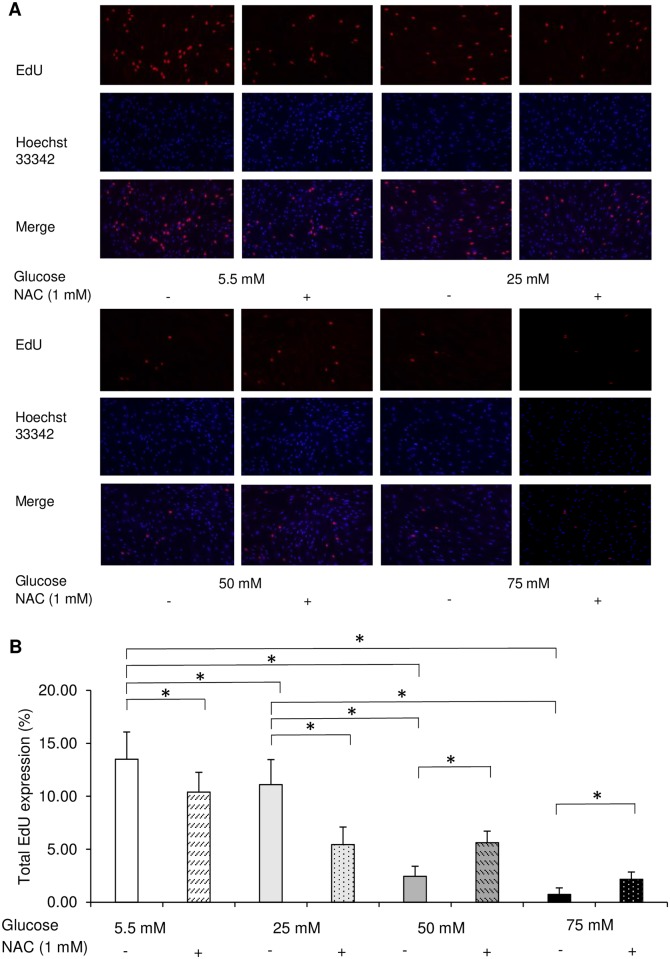
Administration of *N*-acetyl-l-cysteine (NAC) enhanced the proliferation of human gingival fibroblasts. Cell proliferation in the presence of NAC was measured by 5-ethynyl-2′-deoxyuridine (EdU) incorporation assay. (A) Representative immunofluorescence images of EdU incorporated into mitochondrial DNA, Hoechst 33342 staining, and merged images. (B) Quantitative analysis of EdU-positive cells. Data are presented as means ± SD of three independent experiments; mean differences between groups were analyzed using the Tukey–Kramer test. *p < 0.05.

**Fig 8:**
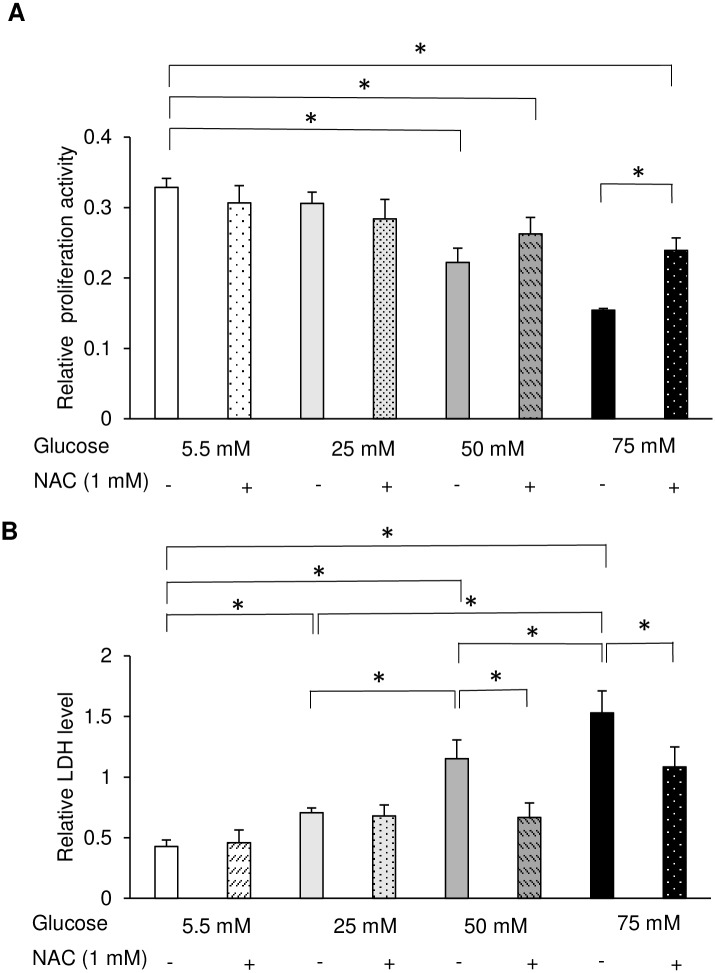
Cell proliferation and cytotoxicity assays in the presence of *N*-acetyl-l-cysteine (NAC). The effects of the antioxidant NAC on the proliferation and cytotoxicity of human gingival fibroblasts (HGFs) were assessed. (A) Results of water-soluble tetrazolium salt (WST-8) proliferation assay of HGFs grown at different glucose and NAC concentrations. (B) Lactate dehydrogenase (LDH) levels in HGFs grown at different glucose and NAC concentrations. Data are expressed as mean values ± SD; mean differences between groups were analyzed using the Tukey–Kramer test. *p < 0.05 compared to values in control group.

To further investigate the involvement of ROS in the functional impairment of HGFs, we next investigated the effect of the antioxidant NAC on HGFs grown at different glucose concentrations. As demonstrated in Fig 6A–6C, NAC significantly restored the cell migration of HGFs at 50 mM glucose. We also performed EdU and WST-8 assays following the administration of 1 mM NAC. Figs 7 and 8A demonstrate the recovery of EdU-positive cell numbers and cell proliferation following the addition of NAC to HGFs grown at 50 and 75 mM glucose. Interestingly, 1 mM NAC inhibited EdU positive cell number in the control (5.5 mM) and 25 mM, while at higher concentrations of NAC (50 and 75 mM), the EdU positive cell number increased. Moreover, it should be noted that the proliferation activity was enhanced, after the application of 1 mM NAC in the 50-mM glucose group, but no significant differences were found. Our findings indicate that the use of 1 mM NAC attenuated the high glucose-induced impairments in HGFs migration and proliferation. These results suggest that high glucose levels inhibit cell migration and proliferation via ROS production in HGFs.

Additionally, to verify whether the cellular damage induced by high glucose could be reversed by NAC, the LDH released from HGFs grown with high glucose and NAC was measured. Application of 1 mM NAC significantly reduced LDH levels in the 50 and 75 mM groups (Fig 8B). These data suggest that the high glucose-induced ROS-impaired functions of HGFs could be recovered by the application of NAC.

## Discussion

Experimental and clinical studies have reported the relationship between periodontal disease and diabetes. Healing and tissue regeneration after periodontal treatment have been found to be diminished in patients with diabetes [14, 20]. A growing body of evidence suggests that high glucose concentrations have deleterious effects on cell function that are dependent on the organ, tissue, and type of cell. For example, 20 mM glucose generates oxidative stress and induces a delay in cell replication in cultured human endothelial cells [21]. In addition, high glucose concentrations of 25 to 50 mM have been found to decrease collagen synthesis in HGFs without affecting cell viability or protein synthesis [9]. It has been reported that glucose levels of 30 and 35 mM cause significant increases in apoptosis in endothelial cells [10] and keratinocytes [11], respectively, but not in fibroblasts. Studies imply that, compared to other cell types, fibroblasts are more resistant to high glucose levels. A recent study reported impaired cell migration in rat gingival fibroblasts after exposure to 75 mM glucose for 72 h and suggested that the optimal glucose concentration for *in vitro* culture of gingival fibroblasts was higher than the optimal concentration *in vivo*. However, culture in 100 mM glucose resulted in the loss of cellular attachment after 9 h in our previous study [7]. This discrepancy can be explained by the fact that the effects of glucose are concentration-dependent and may be distinct in different cell types.

The diagnostic criterion for diabetes is a blood glucose concentration of > 100 mg/dl. Therefore, in the present study, 5.5 mM glucose (100 mg/dl) was used as a control group, and 25, 50, and 75 mM glucose were used as high-glucose groups. To elucidate the effect of high glucose concentrations on the culture of HGFs, cell migration and proliferation were analyzed. In assessing HGFs migration, we found that fibroblasts cultured at 50 and 75 mM glucose showed significant impairments in migration compared to that of the control group. However, long-term wound healing assays (>24 h) cannot distinguish between changes in cell proliferation, cell survival, and cell motility [22]. Since no significant differences in cell proliferation were found during the short-term incubation (6–48 h), the 72 h time point was selected for all further experiments, as shown in S1 Fig. Therefore, we determined cell proliferative activity by measuring DNA synthesis using the EdU assay and confirmed these results with indirect viable cell counting using the WST-8 assay at 72 h; our results were the same as those in scratch assay. Moreover, our results were in agreement with those of other studies showing that high glucose significantly inhibited cell migration [6, 23, 24] and proliferation in fibroblasts [7, 25, 26] from many origins, including the gingiva. In addition to effects on migration and proliferation, the cytotoxicity of high glucose levels was also examined. Significant increases in LDH levels were found in HGFs incubated with 50 and 75 mM glucose compared with levels in the control group. In contrast, incubation with 25 mM glucose did not induce any significant change in LDH levels. Our results are in line with previous investigations in renal cortical fibroblasts, which found a significant increase in apoptosis only when the glucose concentration reached above 33.3 mM [27]. In addition, our findings are in agreement with previous studies of diabetic wounds [28, 29] and fibroblasts from diabetic patients or at high glucose concentrations, which showed decreased cellular migration and proliferation and elevated levels of apoptosis [6, 25, 30]. The presence of a heterogenetic response related to the donor’s age was suspected, but the WST-8 assay showed the same tendency from each subject (S2 Fig).

ROS are formed by free oxygen radicals and produce oxidative stress [31]. High levels of ROS can impair cellular functions such as migration, proliferation, and the extracellular matrix synthesis of fibroblasts and keratinocytes [31]. Research has indicated that hyperglycemia-induced oxidative stress increases ROS formation in a type 2 diabetic mouse model [32] and in rat gingival fibroblasts cultured with high glucose [7], as well as leading to endothelial cell dysfunction [33, 34]. Our investigations showed that the mRNA expression levels of *NRF2*, *HO1*, *SOD1*, and *CAT*, which are markers of oxidative stress, were elevated in the high-glucose groups. Intracellular ROS accumulation in HGFs increased in proportion to increases in glucose concentrations and was significantly higher in the 50 and 75 mM glucose groups after 72 h of incubation. Thus, our data demonstrated that ROS formation was upregulated in HGFs incubated with high glucose (50 and 75 mM). Based on our results and those of previous studies, we speculate that high glucose-induced ROS formation leads to dysfunction in HGFs. Within the limitations of this experiment, the cell number change were not fully taken into consideration in the high-glucose condition with respect to the intracellular ROS data. However, in this study, NAC treatment reversed the inhibited cell number due to the high-glucose condition, and we then concluded that ROS regulated the high-glucose induced inhibition of cell growth at the 72-h time point.

Several reports have shown a marked increase in a number of antioxidant defense mechanisms in diabetes. From studies conducted in various cell types, it appears that NAC promotes cell proliferation [35, 36] and inhibits apoptosis [37, 38]. NAC exerts antioxidant effects by acting as a direct scavenger of free radicals such as OH^•^, H_2_O_2_, and O_2_^-•^ [39]. Interestingly, the concentration at which NAC is effective vary depending on cell type [40]. Our results showed that treatment with NAC at a concentration of 1 mM could restore cell migration and proliferation in HGFs grown at high glucose concentrations, although this NAC concentration had a negative effect on proliferation activity at glucose concentrations of 5.5 and 25 mM. Since normal ROS levels are required to maintain the normal cellular functions, the application of an antioxidant such as NAC, which reduced the amount of intracellular ROS, might disturb the ROS balance required for the physiological roles in the cells [41]. This phenomenon could explain that there exist an optimal concentrations of glucose to culture HGFs. Moreover, 1 mM NAC only partially reversed the cytotoxicity caused by high glucose (50 and 75 mM). It should be noted that the delayed healing process associated with high glucose conditions involves increased oxidative stress in HGFs. Consequently, other factors and pathways may also be involved in the impaired functions of HGFs.

Possible mechanisms underlying the association between periodontal disease and diabetes are currently under investigation and remain controversial. It has been documented that high glucose induced an increase in NF-kB activity [42, 43]. Previous studies have suggested that high glucose-induced ROS generation increases IL-8 production [44], decreases cell migration through inadequate activation of the small Rho GTPase RAC1 [6] and is linked to the inhibition of bFGF signaling, specifically through JNK suppression [23]. Further studies are required to clarify the detailed mechanisms by which hyperglycemia or high glucose conditions affect cell functions like migration and proliferation in periodontal tissue and whether these are mediated by oxidative stress alone or whether insulin resistance or inflammatory gene expression is also involved. Mizutani et al. reported that gingival insulin resistance induced by oxidative stress can affect periodontal repair and destruction, including cell proliferation and angiogenesis [45]. In addition, ROS accumulation is a trigger of the intrinsic death pathway. The earliest evidence implicating ROS in cell death derives from a study of TNF-α-induced cytotoxicity [46]. One possible mechanism by which ROS stimulates apoptosis is through the c-Jun N-terminal kinase (JNK) pathway, which inhibits anti-apoptosis factors like B cell lymphoma-2 (BCL-2) and activates pro-apoptosis factors like Bcl-2-associated X protein (BAX), thereby impairing wound healing [47].

In conclusion, high glucose concentrations impair cell migration and proliferation and cause cell damage in HGFs. Moreover, fibroblast dysfunction may be caused by high glucose-induced oxidative stress. Our *in vitro* conditions were designed to create an environment that mimics diabetic conditions. Gingival fibroblasts are intimately involved in periodontal therapy and are fundamental to the success of surgical procedures. Elevated concentrations of glucose may be a causative factor in the increased incidence of chronic inflammatory periodontitis in diabetes. Thus, our research may serve as a basis for further study into the underlying mechanisms of impaired wound healing in the periodontal tissue of diabetic individuals. Additional experiments are needed, however, to clarify the role of antioxidants such as NAC in order to develop therapeutic approaches for diabetic patients with periodontitis.

## Supporting information

S1 Fig Relative proliferation activity in human gingival fibroblasts (HGFs) following high glucose exposure. WST-8 proliferation assay was performed after (A) 6-h, (B) 24-h, and (C) 48-h culture of HGFs with the indicated concentrations of glucose. No significant differences were found at any time point before 72 h. Data are expressed as mean values ± SD; mean differences between groups were analyzed using the Tukey–Kramer test *p < 0.05 compared to values in control group.
(TIF)

S2 Fig Comparison of proliferative activities of each donor. All the experimental cells used were taken from individual patient and WST-8 proliferation assay was performed. The same tendency from each subject were observed.
(TIF)

S1 Table Primer sequences for PCR. (PDF)
