# Supplementary figures and images for: High glucose-induced oxidative stress impairs proliferation and migration of human gingival fibroblasts
Source: PLoS One. 2018 Aug 9;13(8):e0201855. doi: 10.1371/journal.pone.0201855 (PMC6084939; doi:10.1371/journal.pone.0201855)

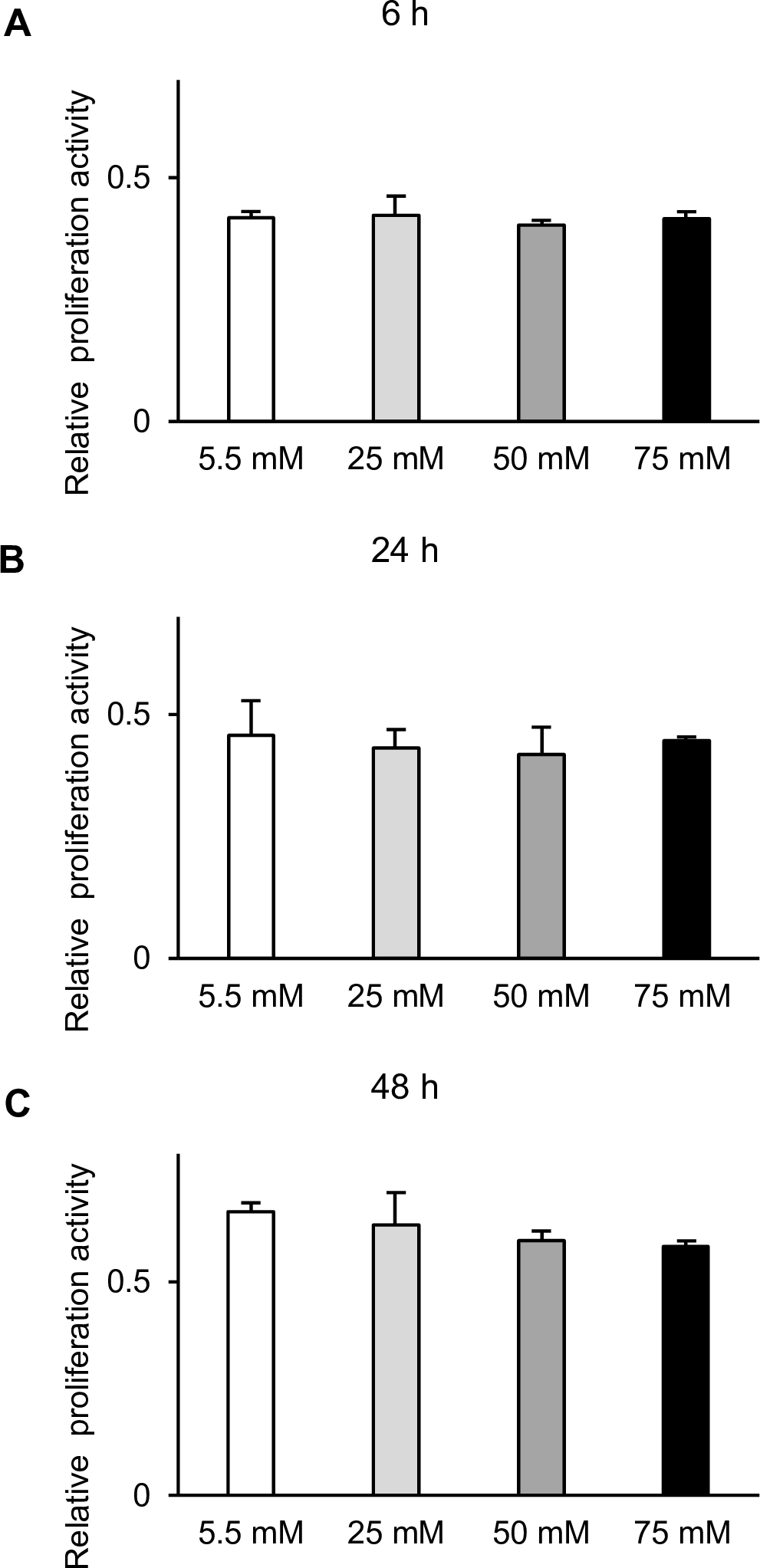

Supplement: S1 Fig — WST-8 proliferation assay was performed after (A) 6-h, (B) 24-h, and (C) 48-h culture of HGFs with the indicated concentrations of glucose. No significant differences were found at any time point before 72 h. Data are expressed as mean values ± SD; mean differences between groups were analyzed using the Tukey–Kramer test *p < 0.05 compared to values in control group. (TIF) [file pone.0201855.s001.tif]

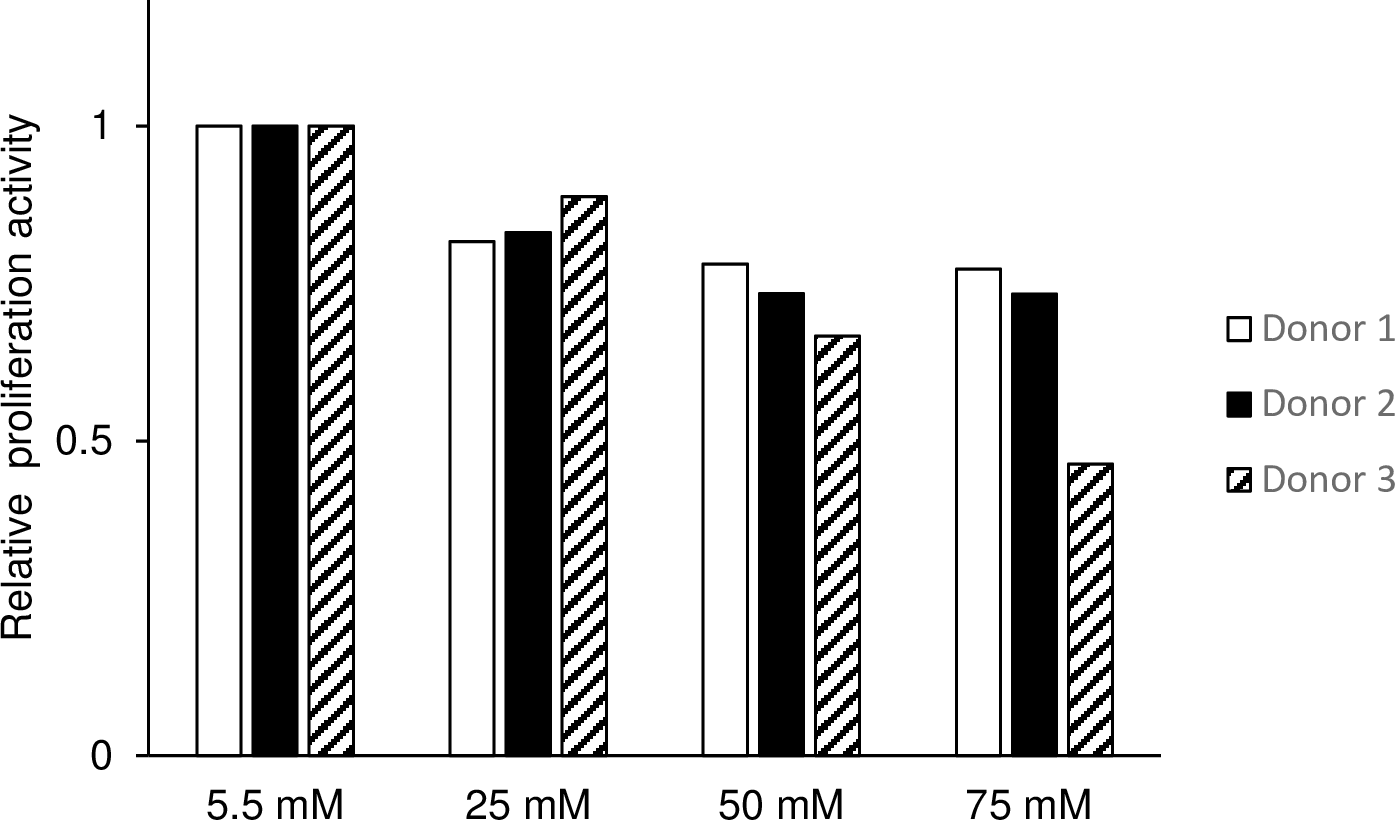

Supplement: S2 Fig — All the experimental cells used were taken from individual patient and WST-8 proliferation assay was performed. The same tendency from each subject were observed. (TIF) [file pone.0201855.s002.tif]
