# Supplementary material for: High glucose-induced oxidative stress impairs proliferation and migration of human gingival fibroblasts
Source: PLoS One. 2018 Aug 9;13(8):e0201855. doi: 10.1371/journal.pone.0201855 (PMC6084939; doi:10.1371/journal.pone.0201855)
Supplement: S1 Table — (PDF) [file pone.0201855.s003.pdf]

Primer sequences used for real-time PCR analysis

Gene	Gen Bank	Forward primer (5'-3')	Reverse primer (5'-3')
NRF2	NM_001313901.1	TTCTCCCAATTCAGCCAGCC	AACGTAGCCGAAGAAACCTCA
HO1	NM_002133.2	ACATCCAGCTCTTTGAGGAGT	TGAGTGTAAGGACCCATCGGA
SOD1	NM_000454.4	CCAGTGCAGGGCATCATCAA	TCTTCATCCTTTGGCCCACC
CAT	NM_001752.3	CGGACATGGTCTGGGACTTC	AACTGCCTCCCCATTTGCAT
β-actin	NM_001101.4	GGCATCCTCACCCTGAAGTA	GGGGTGTTGAAGGTCTCAAA
